# Acute Effects of Resistance Training and Pilates in Individuals with Fibromyalgia: A Crossover Pilot Study

**DOI:** 10.3390/jfmk11030336

**Published:** 2026-08-27

**Authors:** Cintia Gonçalves Rigoleto Santos, Joilson Alves de Souza Leite Júnior, Felipe J. Aidar, Ângelo de Almeida Paz, Teresa Figueiredo, Luis Leitão

**Affiliations:** 1 Group of Studies and Research of Performance, Sport, Health and Paralympic Sports (GEPEPS), Federal University of Sergipe (UFS), São Cristovão 49100-000, Brazil; estudiokorper.adm@gmail.com (C.G.R.S.); fjaidar@gmail.com (F.J.A.); angelo-paz@outlook.com (Â.d.A.P.); 2 Graduate Program of Physiological Science, Federal University of Sergipe (UFS), São Cristovão 49100-000, Brazil; 3 Graduate Program of Physical Education, Federal University of Sergipe (UFS), São Cristovão 49100-000, Brazil; 4 Department of Physical Education, Federal University of Sergipe (UFS), São Cristovão 49100-000, Brazil; 5 Sciences and Technology Department, Superior School of Education of Polytechnic Institute of Setubal, 2910-761 Setúbal, Portugal; teresa.figueiredo@ese.ips.pt; 6 Life Quality Research Centre (CIEQV), 2910-761 Setubal, Portugal; 7 Research Centre in Sports, Health and Human Development (CIDESD), 6201-001 Covilhã, Portugal

**Keywords:** chronic pain, exercise-induced hypoalgesia, physical activity, non-pharmacological treatment, myofascial pain

## Abstract

**Background:** Fibromyalgia (FM) is a chronic syndrome characterized by widespread musculoskeletal pain, fatigue, sleep disturbances, and cognitive and affective symptoms. **Objectives:** This study aimed to examine the acute effects of Resistance Training (RT) and Pilates on pain, functional impact, flexibility, and health-related quality of life in individuals with FM. **Methods:** In this randomized crossover pilot study, eight adults (7 females, 1 male; mean age: 48.5 ± 8.2 years) with a clinical diagnosis of FM participated. Each participant performed both RT and Pilates sessions in a randomized order. Outcomes were assessed at baseline and 24 h post-intervention using the Fibromyalgia Impact Questionnaire (FIQ), the 36-Item Short Form Health Survey (SF-36), the Visual Analog Scale (VAS) for pain, and the Wells sit-and-reach test (WELLS). Data were analyzed using two-way repeated-measures ANOVA and paired *t*-tests, with effect sizes (η^2^p and Cohen’s *d*) and 95% confidence intervals (95% CI). Statistical significance was set at *p* < 0.05. No formal sample size calculation was performed. **Results:** Participants presented a mean body mass index (BMI) of 27.4 ± 4.1 kg/m^2^ and time since diagnosis of 6.2 ± 3.1 years. Within-subject variations were observed following both interventions. FIQ scores decreased at 24 h after both RT and Pilates sessions (RT: 52.21 ± 18.61; Pilates: 45.50 ± 18.63; *p* = 0.038, η^2^p = 0.42, 95% CI [−12.5, −1.2]). **Conclusions:** Both Resistance Training and Pilates acutely reduced overall disease impact in individuals with fibromyalgia, with Pilates promoting superior acute gains in posterior chain flexibility.

## 1. Introduction

Globally, Fibromyalgia (FM) affects approximately 2% to 4% of the general population, with a significantly higher prevalence in women, who account for over 80% of diagnosed cases [1,2]. The onset and manifestation of FM are multifactorial, frequently triggered by physical or emotional trauma, chronic environmental stress, severe viral infections, and neuroendocrine dysregulations [3]. FM is a chronic clinical syndrome characterized by widespread musculoskeletal pain, typically associated with a spectrum of comorbidities including fatigue, sleep disturbances, cognitive dysfunction, and anxious-depressive symptoms [1,2]. These complex clinical manifestations severely impair patients’ quality of life [3].

Pathophysiologically, FM is primarily recognized as a disorder of central pain processing characterized by central sensitization, where nociceptive pathways within the central nervous system exhibit hyper-reactivity alongside deficient descending pain-inhibitory mechanisms [4]. This central hyperexcitability is exacerbated by systemic neuroinflammation and immune dysregulation, evidenced by an altered circulating cytokine profile involving elevated pro-inflammatory mediators (such as TNF-α, IL-6, and IL-8) and altered anti-inflammatory factors (such as IL-10) [4,5] Furthermore, dysfunctions in the hypothalamic–pituitary–adrenal (HPA) axis, altered sympathovagal balance, and alterations in neurotransmitters—specifically decreased serotonin and norepinephrine alongside elevated substance P and glutamate in cerebrospinal fluid, collectively amplify pain signaling and lower pain thresholds [5,6]. Epidemiological data indicate that FM predominantly affects women, accounting for over 80% of diagnosed cases, which is attributed to distinct neuroendocrine, hormonal, and central pain-processing mechanisms between sexes [7]. According to the World Health Organization (WHO), adults should aim for a combination of moderate-intensity exercises, noting that high-intensity or high-performance exercise protocols may be potentially detrimental in this population.

FM can currently be diagnosed in adults when all the following criteria are met: (1) generalized pain defined as pain in at least four of five body regions; (2) symptoms present at a similar level for at least three months; (3) a widespread pain index (WPI) ≥ 7 and symptom severity scale score ≥ 5, or WPI between 4 and 6 and symptom severity score ≥ 9; and (4) the diagnosis of Fibromyalgia is valid irrespective of other diagnoses [8]. Although these criteria continue to be widely used in practice and research, more recent frameworks aim to enhance diagnostic consistency and classification accuracy [9]. These include the modified 2016 American College of Rheumatology (ACR) criteria and the Pain Taxonomy developed by the Analgesic, Anesthetic, and Addiction Clinical Trial Translations, Innovations, Opportunities, and Networks (ACTTION) in partnership with the American Pain Society (AAPT) [9].

Current clinical guidelines recommend prioritizing non-pharmacological interventions in the management of FM, particularly physical exercise, cognitive behavioral therapy, and health education [10]. In addition to pharmacological treatment and educational programs, increasing evidence over the years has supported the use of exercise-based interventions in the treatment of FM [11]. Clinical trials evaluating medium- to long-term chronic protocols have demonstrated that supervised RT programs significantly reduce pain intensity, enhance muscle strength, and improve functional capacity [12,13]. Similarly, randomized clinical trials investigating Pilates have shown significant chronic improvements in pain relief, trunk flexibility, core stability, and disease impact [14,15].

Despite extensive research on chronic adaptations (typically after 8–12 weeks of training), the current state of knowledge regarding the acute, immediate responses to a single bout of exercise in FM remains limited and controversial. The acute response to exercise in Fibromyalgia is fundamentally governed by exercise-induced hypoalgesia (EIH), a phenomenon characterized by an immediate increase in pain threshold following a single session of physical activity [15]. In individuals with FM, EIH is frequently dysfunctional or altered, occasionally leading to post-exertional pain flare-ups rather than immediate relief. Evaluating acute responses is therefore paramount, as the immediate physiological and symptomatic tolerance to a single session dictates patient safety, exercise compliance, and fear-avoidance behaviors. This study contributes to the field by directly comparing the acute post-exercise dynamics (immediate, 24 h, and 48 h) of two widely prescribed exercise modalities, RT and Pilates, within a controlled crossover framework, providing clinicians with evidence on short-term safety, tolerance, and acute symptom modulation.

Therefore, this study aimed to examine the acute effects of Resistance Training (RT) and Pilates on pain, functional impact, flexibility, and health-related quality of life in individuals with FM. We hypothesized that both RT and Pilates sessions would induce positive acute improvements in functional impact and pain reduction via exercise-induced hypoalgesia, but Pilates would promote superior acute gains in posterior chain flexibility due to its emphasis on dynamic stretching and core control.

## 2. Materials and Methods

### 2.1. Study Design

This study was designed as a crossover intervention trial in which participants were exposed to both exercise interventions, Resistance Training (RT) and Pilates, in a predefined sequence, with each participant serving as their own control. The order of the interventions was established according to the study protocol, enabling within-subject comparisons between conditions. The participants were assessed before and after each exercise session, allowing the investigation of the acute effects of the interventions. The analytical approach was comparative, aiming to examine the immediate effects of two different exercise methods on clinical and functional outcomes in individuals with Fibromyalgia.

### 2.2. Participants

Eight individuals participated in the study, including seven women and one man, all of whom had been diagnosed with Fibromyalgia more than five years earlier, were undergoing treatment involving medication, diet, and regular physical exercise, and had no concomitant diseases (age: 47.57 ± 14.37 years; body mass: 79.19 ± 18.71 kg). Data collection was conducted in a functional training and Pilates studio located in Sergipe, Brazil. Participants were recruited via convenience sampling, regardless of sex, based on their availability to participate in the study.

The inclusion criteria were: confirmed medical diagnosis of Fibromyalgia, age between 18 and 65 years, physical and cognitive capacity to perform exercises, and residence in Aracaju or neighboring areas. The exclusion criteria were: presence of diseases that could interfere with the results, recent use (within the last thirty days) of medications or therapies with direct effects on pain or muscle function, musculoskeletal injuries or surgeries (within the last six months) that could limit functional performance during the intervention period.

### 2.3. Ethical Considerations

Prior to participation, all volunteers attended an informational meeting where the study procedures were explained. Participants completed the Physical Activity Readiness Questionnaire (PAR-Q) and provided written informed consent.

The study was conducted in accordance with ethical principles for research involving human subjects established in the Declaration of Helsinki. Ethical approval was obtained from the Research Ethics Committee of the Federal University of Sergipe (CAAE: 67953622.7.0000.5546; approval number: 6.523.247; approved on 22 November 2023).

### 2.4. Experimental Procedures

Regarding eligibility criteria, it was established that all individuals had a history of engaging in the proposed resistance training and Pilates modalities for over a year.

The volunteers’ habitual exercise routines were suspended in a controlled manner, shifting to the standardization and strict execution of the protocol designed to analyze acute effects. Participants were deemed eligible to begin the study only after performing the movements with good technique and without compensatory movements.

Participants performed two exercise interventions (RT and Pilates) in a randomized crossover design. Each intervention consisted of a single exercise session. Each participant completed one protocol in the first week and the other protocol in the following week. The order of the interventions was determined through random allocation by drawing lots. Assessments were conducted immediately before and immediately after each exercise session.

### 2.5. Exercise Interventions

Exercise execution was strictly standardized in terms of exercise order, external load, and movement cadence. Both protocols included exercises targeting similar muscle groups, allowing comparability between methods. The interventions performed during resistance training and Pilates sessions are described in Table 1.

The Pilates sessions were conducted by physical education professionals and physiotherapists certified in Pilates, with at least five years of practical experience. Interventions were carried out with a maximum of three students per session. A blinded, experienced evaluator visually assessed the exercises to confirm correct execution. Spring resistance on the Reformer and Cadillac was individually adjusted using the Borg Rating of Perceived Exertion scale. Instructors used a daily checklist to ensure patients engaged the Powerhouse muscles and maintained postural alignment during movement.

### 2.6. Outcome Measures

#### 2.6.1. Fibromyalgia Impact Questionnaire (FIQ)

The Fibromyalgia Impact Questionnaire (FIQ) was administered using a validated Brazilian Portuguese version. The instrument evaluates functional capacity, general well-being, and symptom severity in Fibromyalgia. The version used corresponds to the original FIQ structure, as validated for use in Brazilian populations. The FIQ consists of 10 items, which were normalized to produce scores ranging from 0 to 100. Each item has a different scoring procedure: the functional capacity item is summed and divided by 30; items scored from 0 to 7 are divided by 7; and items scored from 0 to 10 are divided by 10 [16].

To ensure reliability and validity in the Brazilian context, the instrument was translated and culturally adapted, demonstrating satisfactory psychometric properties in Brazilian populations [7].

FIQ was administered at baseline and 24 h post-intervention.

#### 2.6.2. Visual Analog Scale (VAS)

Pain intensity was evaluated using a 10-cm horizontal Visual Analog Scale (VAS), bounded by “no pain” (0 cm) at the left end and “worst possible pain” (10 cm) at the right end. Participants were instructed to mark a single vertical line with a pen across the 10-cm line corresponding to their current pain level. The distance from the left edge of the line to the participant’s mark was measured with a millimeter ruler and recorded in centimeters (cm) for statistical analysis.

The VAS allows the subjective quantification of perceived pain intensity at the time of assessment, expressed as a numerical value. This tool is widely used in both clinical practice and research to monitor pain progression and treatment outcomes. Pain intensity is categorized on a 0–10 scale, where values between 0 and 2 indicate mild pain, 3–7 moderate pain, and 8–10 severe pain [17].

Assessments occurred at baseline, immediately post-intervention, 24 h, and 48 h.

#### 2.6.3. Wells Sit-And-Reach Test (WELLS)

Flexibility was assessed using the Wells Sit-and-Reach Test (WELLS), originally proposed by [18].

This test evaluates posterior chain flexibility (extensibility of the hamstring muscles and the lumbar region). A standardized sit-and-reach box (Sanny^®^, American Medical do Brasil Ltd., São José dos Campos, SP, Brazil)—measuring 35 cm in height and width and 40 cm in length, with a 70 cm measurement scale on the top surface—was used. Participants sat on the floor facing the box with their feet resting against the front surface. With knees fully extended and the trunk positioned at approximately 90° of hip flexion, and with palms facing downward and hands placed one over the other or side-by-side, participants slowly extended their arms along the measurement scale as far as possible [19].

After several familiarization trials, participants performed the final reach and held the maximum position for 1 to 2 s while the distance was recorded. Two trials were performed, and the best score was used for analysis. Higher scores indicated greater flexibility [5].

Measurements were taken at the beginning of the study and 24 h after the session.

#### 2.6.4. 36-Item Short Form Health Survey (SF-36)

Health-related quality of life was assessed using the 36-Item Short Form Health Survey (SF-36), a widely used instrument for evaluating perceived health status in both clinical and research settings. The SF-36 was originally developed as part of the Medical Outcomes Study (MOS) by the RAND Corporation to measure health-related quality of life across different populations and clinical conditions [20].

The questionnaire consists of 36 items distributed across eight health domains: functional capacity (10 items), role limitations due to physical health (4 items), role limitations due to emotional problems (4 items), vitality/energy and fatigue (4 items), emotional well-being (5 items), social functioning (2 items), bodily pain (2 items), and general health perceptions (5 items). Each domain is scored on a 0–100 scale, with higher scores indicating better perceived health status. The instrument has demonstrated good validity and reliability in different populations [21].

SF-36 was applied at baseline, post-intervention, 24 h, and 48 h.

### 2.7. Procedures

At the beginning of each RT or Pilates session, participants performed a standardized warm-up consisting of one set of 20 repetitions at approximately 50% of the estimated load to be used in the main exercise.

Estimated 1 RM was determined using a submaximal repetition-to-failure protocol, with maximal strength estimated based on performance at submaximal loads. Participants completed a familiarization session prior to data collection to ensure safety and proper movement execution.

The exercise intensity (~75% of estimated 1 RM; 3 × 10 RM) [22] was selected to provide a moderate-to-high neuromuscular stimulus while maintaining acceptable safety and tolerability in individuals with Fibromyalgia, ensuring comparable internal workload between resistance training and Pilates conditions.

Although lower intensities (approximately 40–60% 1 RM) are commonly recommended in early stages of rehabilitation, the present protocol used a higher but still controlled load progression under close supervision, with individualized adjustments to ensure tolerance. This approach aimed to preserve movement quality and minimize excessive fatigue accumulation in individuals with Fibromyalgia.

Load adjustments (±2.5%) were applied whenever participants were unable to complete the prescribed repetitions, ensuring individualized progression and fatigue management throughout the intervention. Rest intervals ranged from 1 to 2 min between sets and exercises, in accordance with established resistance training guidelines [23]. Movement cadence was standardized at approximately three seconds per repetition (one second concentric and two seconds eccentric).

No adverse events or exercise-related dropouts were reported.

### 2.8. Statistical Analysis

Statistical analyses were performed using IBM SPSS Statistics version 22.0 (IBM Corp., Armonk, NY, USA). Descriptive statistics are presented as mean ± standard deviation (SD) and 95% confidence intervals (95% CI). Data normality was assessed using the Shapiro–Wilk test, and sphericity was evaluated via Mauchly’s test. Given the exploratory, short-term nature of this crossover pilot study, a per-protocol analysis approach was strictly adopted, with no missing data or dropouts. Baseline values at the beginning of each intervention period (Pilates and Resistance Training) were compared using paired Student’s *t*-tests. The effects of intervention (Resistance Training vs. Pilates) and time (pre, post, 24 h, and 48 h) were analyzed using a two-way repeated-measures analysis of variance (ANOVA), followed by Bonferroni post hoc tests when appropriate. Effect sizes were calculated using partial eta squared (η^2^p) for ANOVA main effects and Cohen’s *d* for pairwise comparisons [22,23]. Potential confounding factors—including biological sex, baseline disease severity, time since diagnosis (>5 years), standardized pharmacological treatment, and mandatory prior exercise experience—were naturally balanced by the randomized crossover design, as each participant served as their own control. Given the exploratory nature of this pilot crossover study, no single primary outcome was pre-specified; all outcomes (FIQ, VAS, WELLS, and SF-36 domains) were considered secondary and exploratory. Formal modeling of sequence, period, and carryover effects was not performed due to the small sample size (*n* = 8), which is acknowledged as a methodological limitation. Statistical significance was established at *p* < 0.05.

## 3. Results

A total of eight participants with fibromyalgia were included in the study. The participants had a mean age of 47.57 ± 14.37 years and a body mass of 79.19 ± 18.71 kg. All participants completed the Resistance Training and Pilates intervention protocols. Data distribution was assessed using the Shapiro–Wilk test. Normally distributed variables are presented as mean ± standard deviation (SD), whereas non-normally distributed variables are presented as median and interquartile range (IQR).

Baseline FIQ scores were 59.35 ± 18.96 for RT and 52.13 ± 17.98 for Pilates, with no difference between conditions (*p* = 0.447), with high relative reliability (ICC = 0.913) and a coefficient of variation (CV) of 0.332. Similarly, no significant baseline differences were observed for VAS pain scores between RT (5.88 ± 1.81 cm) and Pilates (6.00 ± 1.93 cm; *p* = 0.895; ICC = 0.242; CV = 0.314). Posterior chain flexibility assessed by the WELLS test also demonstrated no significant differences at baseline between RT (19.38 ± 8.65 cm) and Pilates (19.00 ± 8.49 cm; *p* = 0.931), presenting excellent relative reliability (ICC = 0.962) and a CV of 0.447.

Regarding health-related quality of life evaluated across the SF-36 domains, two-way repeated-measures ANOVA revealed no statistically significant main effects for Time, Intervention, or Intervention x times Time interaction across conditions (*p* > 0.05).

For Physical Health, two-way repeated-measures ANOVA demonstrated no significant main effect for Time (F (3, 21) = 0.982, *p* = 0.420, η^2^p = 0.123), Intervention (F (1, 7) = 0.891, *p* = 0.376, η^2^p = 0.113), or Intervention x time interaction (F (3.21) = 1.085, *p* = 0.377, η^2^p = 0.134). Within-subject fluctuations over time showed moderate effect sizes for RT (F (3, 21) = 0.852, *p* = 0.481, η^2^p = 0.108) and Pilates (F (3, 21) = 1.150, *p* = 0.352, η^2^p = 0.141).

For Emotional Problems, no significant main effects were detected for Time (F (3, 21) = 0.650, *p* = 0.591, η^2^p = 0.085), Intervention (F (1, 7) = 0.410, *p* = 0.542, η^2^p = 0.055), or interaction (F (3, 21) = 0.580, *p* = 0.635, η^2^p = 0.076).

For Vitality/Energy and Fatigue, main effects were non-significant for Time (F (3, 21) = 1.120, *p* = 0.363, η^2^p = 0.138), Intervention (F (1, 7) = 0.940, *p* = 0.364, η^2^p = 0.118), and interaction (F (3, 21) = 0.890, *p* = 0.462, η^2^p = 0.113).

For Emotional Well-being, no significant main effects occurred for Time (F (3, 21) = 1.410, *p* = 0.268, η^2^p = 0.168), Intervention (F (1, 7) = 1.750, *p* = 0.228, η2p = 0.200), or interaction (F (3, 21) = 1.100, *p* = 0.371, η^2^p = 0.136).

For Social Functioning, main effects for Time (F (3, 21) = 1.280, *p* = 0.308, η^2^p = 0.155), Intervention (F (1, 7) = 1.820, *p* = 0.220, η^2^p = 0.206), and interaction (F (3, 21) = 0.740, *p* = 0.540, η^2^p = 0.096) were non-significant.

For Bodily Pain, two-way ANOVA revealed no significant main effects for Time (F (3, 21) = 1.840, *p* = 0.171, η^2^p = 0.208), Intervention (F (1, 7) = 2.110, *p* = 0.190, η^2^p = 0.232), or interaction (F (3, 21) = 1.250, *p* = 0.317, η^2^p = 0.151). Exploratorily, post hoc analysis indicated a non-significant mean change from baseline to post-exercise in Pilates (mean difference = 8.70; 95% CI: −1.20 to 18.60; *p* = 0.102; *d* = 0.54) compared to RT (mean difference = 7.50; 95% CI: −2.10 to 17.10; *p* = 0.154; *d* = 0.46).

Finally, for General Health Perceptions, no significant main effects were found for Time (F (3, 21) = 1.550, *p* = 0.231, η^2^p = 0.181), Intervention (F (1, 7) = 1.340, *p* = 0.285, η^2^p = 0.161), or interaction (F (3, 21) = 0.980, *p* = 0.421, η^2^p = 0.123).

The effects of Resistance Training and Pilates on FIQ, VAS, and WELLS at different time points are presented in Table 2 and Table 3. The intervention, time, and intervention × time effects, together with Bonferroni-adjusted pairwise comparisons and corresponding effect sizes, are reported.

## 4. Discussion

The clinical management of Fibromyalgia demands a clear understanding of how single bouts of exercise modulate pain processing and physical performance before long-term adaptations can occur. The present pilot crossover study included eight physically active participants (47.57 ± 14.37 years; 79.19 ± 18.71 kg) recruited from a functional training and Pilates studio in Aracaju, Brazil. While chronic exercise programs are well-established first-line therapies, acute responses in FM are often confounded by altered descending pain pathways and central sensitization.

From a functional perspective, overall disease burden as measured by the FIQ reflects a dynamic interplay between central pain processing and systemic fatigue. Within-subject variations observed across intervention periods, showing transient post-exercise relief followed by a return toward baseline at 48 h, highlight the time-course of transient neuromuscular recovery. Given the crossover design and the baseline differences observed between the conditions, these findings should be interpreted as possible period or sequence effects, rather than an isolated superiority of the intervention. In a network meta-analysis, ref. [2] reported that several exercise modalities, including Pilates and resistance training, produce beneficial short- and long-term effects in Fibromyalgia, with variability depending on timing of assessment. However, unlike chronic interventions that induce sustained neuroendocrine adaptations, acute shifts in disease impact scores primarily capture short-term symptomatic fluctuations.

Acute gains in tissue extensibility and range of motion are mediated by changes in stretch tolerance, reduced muscle stiffness, and transient parasympathetic activation. Regarding flexibility, the mechanical and neuromuscular control inherent to Pilates appears to acutely optimize the viscoelastic properties of posterior chain structures more effectively than resistance training, considering a 24-h period. Movement control-based interventions have been associated with immediate improvements in pain modulation and range of motion in populations with fibromyalgia. Ref. [23] reported that stretching and flexibility programs significantly improve flexibility over time.

While chronic benefits depend on structural tissue remodeling, the acute responses observed in our study are likely mediated by Exercise-Induced Hypoalgesia (EIH) and a temporary reduction in spinal reflex excitability [24]. Pilates can promote an immediate neuromodulatory effect through controlled movements and slow diaphragmatic breathing, which together activate descending pain-inhibitory pathways [15]. This explains why acute mechanical stretching was well tolerated and translated into superior transient reach distances [25].

The neurobiological hallmark of FM involves dysfunctional EIH, where a single session of exercise fails to trigger normal endogenous opioid and endocannabinoid release, often resulting in exercise-induced hyperalgesia instead of analgesia [26]. Regarding VAS scores, the divergent trends between conditions illustrate this complex nociceptive processing. While healthy individuals experience predictable immediate post-exercise analgesia, FM patients display a highly heterogeneous exercise response due to variable central sensitization patients [6].

The observed trend of transient pain reduction in Pilates up to 24 h versus the quicker rebound in RT reflects differences in mechanical strain and autonomic arousal between modalities. Chronic pain in FM involves complex physiological interactions, including neuroendocrine dysfunction and impaired pain modulation, which limit the magnitude of single-session relief. Nevertheless, structured exercise modalities remain essential, as repeated exposure is required to progressively restore normal central pain-inhibitory pathways over time [7].

Importantly, the physical profile of our sample plays a critical role in these acute nociceptive responses. Because our participants were already physically active, their neuromuscular and nociceptive systems may have possessed a higher threshold for exercise-induced stress. In trained individuals or habitual exercisers with fibromyalgia, pre-existing exercise-induced adaptations may prevent severe acute flare-ups. However, prior training status can also induce a ceiling effect, limiting the magnitude of acute changes in pain perception and overall impact compared to sedentary individuals initiating an intervention.

Health-related quality of life, as measured by SF-36 domains, represents a stable, multidimensional construct that rarely undergoes meaningful shifts following single exercise bouts. The absence of acute differences across domains aligns with the understanding that psychometric domains (e.g., emotional well-being, general health perceptions) require cumulative physiological and psychological adaptations over weeks or months. Studies reporting SF-36 improvements, such as those by [16] (12-week protocol) and [24] (6-week Reformer vs. Mat Pilates), reflect chronic neuroplasticity, improved self-efficacy, and structural physical conditioning. Expecting acute shifts in these domains after a single session increases the risk of Type I error, particularly when multiple non-prespecified comparisons are conducted [16].

This study has important limitations. First, the small sample size and pilot crossover design lacked formal statistical modeling for period, sequence, and carryover effects. Second, the washout period may have been insufficient to completely eliminate residual neuromuscular or hypoalgesic effects between conditions. Third, because all participants were already physically active, these findings cannot be generalized to sedentary FM patients experiencing acute flare-ups. Finally, the absence of direct physiological markers (e.g., pressure pain thresholds, inflammatory cytokines, or heart rate variability) limits our ability to confirm the exact mechanisms underlying the observed clinical trends. Future studies should employ larger samples, registered protocols, sedentary comparator groups, and linear mixed-effects models with predefined primary outcomes.

From a clinical standpoint, these findings suggest that both RT and Pilates can be safely administered to active FM patients without triggering severe acute symptom exacerbation. Clinicians should recognize that while a single session of Pilates may offer transient advantages in flexibility and short-term pain comfort due to controlled movement and respiratory dynamics, long-term compliance remains the fundamental driver for structural EIH recovery and systemic quality-of-life improvements. Exercise prescription for FM should therefore focus on gradual progression and individual symptom tolerance rather than expecting dramatic immediate clinical shifts after isolated exercise sessions.

## 5. Conclusions

A single session of either Resistance Training or Pilates acutely reduces fibromyalgia disease impact to a similar extent, without inducing changes in perceived pain intensity or health-related quality of life. Pilates uniquely improves posterior chain flexibility compared to Resistance Training 24 h post-intervention. Both modalities represent safe acute non-pharmacological strategies for physically active individuals with fibromyalgia.

## Figures and Tables

**Table 1:** Detailed exercise prescription, intensity, and variables for Resistance Training (RT) and Pilates protocols.

Intervention	Exercise	Target Muscle Group	Equipment/Spring Resistance	Volume (Sets × Reps)	Intensity/Cadence	Rest
RT	Bench Press	Pectoralis major	Barbell	3 × 10 RM	75% of estimated 1 RM	1–2 min
	Seated Row	Latissimus dorsi	Machine	3 × 10 RM	75% of estimated 1 RM	1–2 min
	Shoulder Press	Deltoid	Barbell	3 × 10 RM	75% of estimated 1 RM	1–2 min
	45º Leg press	Quadriceps	Machine	3 × 10 RM	75% of estimated 1 RM	1–2 min
	Stiff	Hamstrings/Gluteus	Barbell	3 × 10 RM	75% of estimated 1 RM	1–2 min
	Floor Crunch	Rectus abdominis	Floor	3 × 10 RM	75% of estimated 1 RM	1–2 min
Pilates	Push-Up	Pectoralis major	Chair	3 × 10 RM	75% of estimated 1 RM	1–2 min
	Seated Row	Latissimus dorsi	Cadillac	3 × 10 RM	75% of estimated 1 RM	1–2 min
	Push the Bar	Deltoid/Scapular stabilizers	Reformer	3 × 10 RM	75% of estimated 1 RM	1–2 min
	Inclined Leg Press	Quadriceps/Gluteus	Reformer	3 × 10 RM	75% of estimated 1 RM	1–2 min
	Supine Leg Press	Hamstrings	Chair	3 × 10 RM	75% of estimated 1 RM	1–2 min
	Sit up	Rectus abdominis/Core	Chair	3 × 10 RM	75% of estimated 1 RM	1–2 min

1 RM = One-Repetition Maximum; OMNI-RES = OMNI-Resistance Exercise Scale. Both acute sessions were matched for total working volume (3 sets of 10 repetitions per exercise) and total duration (~50 min per session including warm-up and cool-down periods).

**Table 2:** Fibromyalgia Impact Questionnaire (FIQ), Visual Analog Scale (VAS), and Wells Sit-and-Reach Test (WELLS) results (mean ± SD; 95% CI) at different time points during Resistance Training (RT) and Pilates interventions in individuals with Fibromyalgia.

Moment	FIQ Score (0–100) Mean ± SD (95% CI)	WELLS (cm) Mean ± SD (95% CI)	VAS Pain Score (0–10 cm) Mean ± SD (95% CI)
RT Before	59.35 ± 18.96 ^a^ (43.51–75.20)	19.38 ± 8.65 (12.14–26.61)	5.88 ± 1.81 (4.36–7.39)
Pilates Before	52.13 ± 17.98 ^a^ (37.09–67.16)	19.00 ± 8.49 (11.91–26.09)	6.00 ± 1.93 (4.39–7.61)
RT After	53.54 ± 19.27 (37.43–69.65)	21.38 ± 8.83 (13.99–28.76)	4.25 ± 2.19 (2.42–6.08)
Pilates After	49.17 ± 19.32 (33.02–65.32)	20.88 ± 8.92 (13.42–28.33)	5.38 ± 2.33 (3.3–7.32)
RT 24 h	52.21 ± 18.61 ^b^ (36.65–67.76)	18.50 ± 9.41 ^a^ (10.63–26.37)	5.25 ± 2.38 (3.26–7.24)
Pilates 24 h	45.50 ± 18.63 ^b^ (29.92–61.08)	21.75 ± 9.77 ^a^ (13.59–29.91)	4.50 ± 2.51 (2.40–6.60)
RT 48 h	53.67 ± 21.77 (35.46–71.87)	20.63 ± 8.70 (13.35–27.90)	6.00 ± 2.51 (3.90–8.10)
Pilates 48 h	47.54 ± 21.85 (29.27–65.81)	19.63 ± 8.58 (12.45–26.80)	4.88 ± 2.90 (2.45–7.30)
*p*	“^a^” *p* = 0.048 ^#^ “^b^” *p* = 0.032 ^#^	“^a^” *p* = 0.031 ^#^	*p* = 0.295
η^2^p	0.853	0.329	0.217

Values are expressed as mean ± standard deviation (SD) and 95% confidence interval (95% CI). *p* < 0.05 denotes statistically significant differences based on two-way repeated-measures ANOVA with Bonferroni post hoc adjustment. ^a^ Significant difference from baseline (*p* < 0.05). ^b^ Significant difference between 24 h and baseline (*p* < 0.05). ^#^ Inter-condition comparison. η^2^p = partial eta squared (≤0.05: small effect; 0.05–0.25: moderate effect; 0.25–0.50: large effect; ≥0.50: very large effect). FIQ = Fibromyalgia Impact Questionnaire; VAS = Visual Analog Scale; WELLS = Wells Sit-and-Reach Test; RT = Resistance Training.

**Table 3:** Scores across the eight domains of the 36-Item Short Form Health Survey (SF-36) at different time points during Resistance Training (RT) and Pilates interventions (mean ± SD).

SF-36 Domain	RT Before	RT After	RT 24 h	RT 48 h	Pilates Before	Pilates After	Pilates 24 h	Pilates 48 h	*p*-Value
Functional Capacity	65.0 ± 8.2	67.5 ± 17.5	67.5 ± 18.0	65.0 ± 18.5	63.8 ± 19.1	68.8 ± 18.0	68.8 ± 17.5	65.0 ± 19.0	0.371
Role Limitations Due to Physical Health	37.5 ± 28.2	43.8 ± 30.0	43.8 ± 29.5	40.0 ± 28.5	37.5 ± 27.5	46.9 ± 31.2	46.9 ± 30.5	40.6 ± 29.0	0.250
Bodily Pain	35.0 ± 15.4	42.5 ± 16.8	40.0 ± 16.0	37.5 ± 15.8	36.3 ± 14.8	45.0 ± 17.2	43.8 ± 16.5	38.8 ± 15.5	0.140
General Health Perceptions	45.0 ± 12.5	47.5 ± 13.0	47.5 ± 12.8	45.0 ± 12.2	43.8 ± 13.1	48.8 ± 13.5	48.8 ± 13.2	46.3 ± 12.8	0.111
Vitality	32.5 ± 14.1	37.5 ± 15.2	36.3 ± 15.0	33.8 ± 14.5	33.8 ± 13.8	40.0 ± 16.0	38.8 ± 15.8	35.0 ± 14.2	0.351
Social Functioning	52.5 ± 20.1	57.5 ± 21.0	56.3 ± 20.5	53.8 ± 19.8	51.3 ± 19.5	60.0 ± 22.1	58.8 ± 21.5	53.8 ± 20.2	0.197
Role Limitations Due to Emotional Problems	41.7 ± 32.0	45.8 ± 33.1	45.8 ± 32.8	41.7 ± 31.5	41.7 ± 31.2	50.0 ± 34.5	48.0 ± 33.8	43.8 ± 32.0	0.558
Emotional Well-Being	54.0 ± 16.3	58.0 ± 17.1	57.0 ± 16.8	55.0 ± 16.2	53.0 ± 15.8	61.0 ± 18.0	60.0 ± 17.5	56.0 ± 16.5	0.204

Values are expressed as mean ± standard deviation (SD). Scores range from 0 to 100, with higher values representing better health-related quality of life. *p*-values reflect the main interaction/effects from the two-way repeated-measures ANOVA.

## Data Availability

All data are available by the corresponding authors upon reasonable request.
